# Overexpression of *β*-Catenin Induces Cisplatin Resistance in Oral Squamous Cell Carcinoma

**DOI:** 10.1155/2016/5378567

**Published:** 2016-07-27

**Authors:** Long Li, Hai-Chao Liu, Cheng Wang, Xiqiang Liu, Feng-Chun Hu, Nan Xie, Lanhai Lü, Xiaohua Chen, Hong-Zhang Huang

**Affiliations:** ^1^Department of Oral and Maxillofacial Surgery, Guanghua School and Hospital of Stomatology, Sun Yat-sen University, Guangzhou 510055, China; ^2^Guangdong Key Laboratory of Stomatology, Sun Yat-sen University, Guangzhou 510074, China; ^3^Department of Oral Pathology, Guanghua School and Hospital of Stomatology, Sun Yat-sen University, Guangzhou 510055, China

## Abstract

Abnormal expression of *β*-catenin contributes to tumor development, progression, and metastasis in various cancers. However, little is known about the relationship between abnormal expression of *β*-catenin and cisplatin chemotherapy in oral squamous cell carcinoma (OSCC). The present study aimed to investigate the effect of *β*-catenin on OSCC cisplatin resistance and evaluated the drug susceptibility of stable cell lines with *β*-catenin knockin and knockdown. In this study, we found that higher expression level of *β*-catenin can be observed in CDDP-treated cell lines as compared with the control group. Furthermore, the expression levels of *β*-catenin increased in both a concentration- and time-dependent manner with the cisplatin treatment. More importantly, the nuclear translocation of *β*-catenin could also be observed by confocal microscope analysis. Stable cell lines with CTNNB1 knockin and knockdown were established to further investigate the potential role and mechanism of *β*-catenin in the chemoresistance of OSCC in vitro and in vivo. Our findings indicated that overexpression of *β*-catenin promoted cisplatin resistance in OSCC in vitro and in vivo. We confirmed that GSK-3*β*, C-myc, Bcl-2, P-gp, and MRP-1 were involved in *β*-catenin-mediated drug resistance. Our findings indicate that the Wnt/*β*-catenin signaling pathway may play important roles in cisplatin resistance in OSCC.

## 1. Introduction

Head and neck cancer is the sixth most common cancer worldwide with approximately 600,000 new cases per year [1]. Between 40% and 60% of the patients with locally advanced head and neck cancer will relapse [2, 3], and nearly 50% of patients will die due to tumor-related complications [4, 5]. The vast majority of the head and neck cancers are oral squamous cell carcinomas (OSCC). The incidence of OSCC has been rapidly increasing in Asia [6] and more than 12% of these cases occur in China [7]. Furthermore, the number of deaths of patients with tongue squamous cell carcinoma has increased by 11% during the past 5 years [8, 9]. Conventional treatments for OSCC include surgery, radiotherapy, and chemotherapy. Although treatments have greatly improved in the past few years, the survival rate in patients with advanced OSCC treated by surgery alone or radiotherapy alone is still poor. Compared with a single modality treatment, a combination of surgery, radiotherapy, and chemotherapy increases the 5-year overall survival rate by 6.5% [10]. Patients receiving chemotherapy had better survival rates than those not receiving chemotherapy [11, 12]. Chemotherapy has several benefits, especially for patients being considered for organ-preserving treatment, including reduced metastasis, improved local regional control, longer survival, and preservation of organ function [13]. Chemotherapy in conjunction with surgery or radiotherapy was more effective in advanced OSCC [14, 15]. However, the effectiveness of chemotherapy is limited by chemoresistance to certain anticancer drugs [16]. Cisplatin is a first-generation anticancer compound that has been widely used to treat OSCC for many years. However, cisplatin used in the chemotherapy for OSCC often fails because of the rapid development of both inherent and acquired chemoresistance, which limits its application [17]. The molecular mechanisms of cisplatin drug resistance in OSCC remain largely unknown.

*β*-catenin, coded by CTNNB1, is an important adhesion molecule and a key regulator in the Wnt signaling pathway, which has many important functional roles including the maintenance of epithelial stability, growth, and differentiation [18]. The Wnt/*β*-catenin pathway was reported to be associated with chemoresistance in epithelial ovarian cancer [19, 20]. Furthermore, *β*-catenin has been demonstrated to be associated with drug resistance in different types of human cancers [21, 22]. However, little is known about the relationship between drug resistance and *β*-catenin in OSCC.

In our previous study, we demonstrated that P-glycoprotein (P-gp), Bcl-2, MDR, and multidrug resistance-associated proteins-1 (MRP-1) were involved in cisplatin resistance in tongue squamous cell carcinoma [23]. We confirmed that there was abnormal expression of *β*-catenin in tissue samples from patients who had received cisplatin chemotherapy compared with tissue specimens from patient without chemotherapy. We found that overexpression of *β*-catenin could promote P-gp, Bcl-2, MDR, and MRP-1 expressions that enhanced cisplatin resistance in OSCC cell lines. These findings provided important clues to the mechanisms involved in OSCC drug resistance. In addition, we found an increase in the expression levels of *β*-catenin in the OSCC cell lines treated with cisplatin compared with the controls. Therefore, we hypothesize that *β*-catenin may play a crucial role in the development of drug resistance in OSCC. In the present study, we investigated the role of *β*-catenin in the development of chemoresistance in OSCC cell lines.

## 2. Materials and Methods

### 2.1. Cell Culture and Treatment

Two human OSCC cell lines (SCC-15 and SCC-25) were selected for analysis. SCC-15 and SCC-25 cells (American Type Culture Collection, USA) were cultured in DMEM-Ham's F12 medium (Gibco, USA) with 400 ng/mL hydrocortisone (Sigma, USA), 1% penicillin/streptomycin (Gibco, USA), and 10% FBS (Hyclone Laboratories Inc., UT, USA). All cells were incubated in a humidified atmosphere containing 5% CO_2_ at 37°C and then subcultured by trypsinization (Trypsin-EDTA, Lonza). For the analysis of the expression levels of *β*-catenin, OSCC cells were treated with cisplatin (0.5 and 2 *μ*g/mL) for 24 h. The control cells were treated with regular media.

### 2.2. Establishment of Stable OSCC Cell Lines with CTNNB1 Knockin and Knockdown

We used recombinant lentiviruses, pGLV3-GFP-CTNNB1-shRNA, pGLV3-GFP-NC, pGLV5-GFP-CTNNB1, and pGLV5-GFP-NC (Vipotion Biotechnology, Guangzhou, China). The amplification primer sequence for CTNNB1: CTNNB1-F: 5′-ATGGCTA-CTCAAGCTGATTTGAT-3′, CTNNB1-R: 5′-TTACAGGTCAGTATCAAACCA-GG-3′. The siRNA primer sequences are siRNA-homo-1302, 5′-AGGTGCTAT-CTGTCTGCTCTA-3′, and NC siRNA, 5′-TTCTCCGAACGTGTCACGTTTC-3′.

Stable SCC-25 cell lines with CTNNB1 knockin and knockdown were established via infection of the high titer lentiviral particles (GenePharma, Shanghai, China), which was cotransfected into 293T cells and purified according to the manufacturer's instructions. The supernatant containing infectious lentiviruses was harvested for transductions. The isolated transfectants were further selected by culture in 2 mg/mL Puromycin (Life Technologies, USA) and confirmed by proliferation assay. The transfection efficiency was detected by PCR and Western blotting. Stable expressing cells with CTNNB1 knockin and knockdown were used in the subsequent study.

### 2.3. Cell Apoptosis Assay

For the apoptosis assay, cells with stable CTNNB1 overexpression and knockdown were treated with 2 *μ*g/mL cisplatin (Sigma, USA) for 24 h following the manufacturer's protocol. Cells were collected and stained with Annexin V PE/7AAD apoptosis kit (LiankeBio, Hangzhou, China) and detected by flow cytometry (FACSCalibur, BD Biosciences, USA). Cells that were negative with propidium iodide (PI) and Annexin V are considered healthy, cells are considered apoptotic with PI negative and Annexin V positive, and cells that are positive to both PI and Annexin V are considered necrotic. The experiments were repeated at least three times.

### 2.4. Assessment of Cell Viability and Proliferation

Cells with stable CTNNB1 overexpression and knockdown were seeded at a density of 5.0 × 10^3^ cells per well for cell the viability assay and 2.0 × 10^3^ cells per well for the proliferation assay and were cultured accordingly. For the cell viability assay, cells were treated with cisplatin (0, 0.5, 1, 2, 4, 8, and 16 *μ*g/mL) for 24 h. Proliferation assay was performed on days 1 to 7. Cell growth was assessed using the Cell Counting Kit 8 assay (CCK-8; Beyotime Biotechnology, China) according to the manufacturer's protocol. After appropriate incubation, 10 *μ*L of CCK-8 solution was added per well and incubated at 37°C for 1 hour. The absorbance of each well was measured at 450 nm using a microplate reader (Multiskan MS; Thermo Fisher Scientific, USA). Growth curve was drawn to assess the effect of *β*-catenin on proliferation. The cytotoxicity of cisplatin in the OSCC cell lines was quantified by the inhibitory concentration 50% (IC_50_), which was calculated as previously described [24]. Each experiment was repeated at least three times.

### 2.5. Confocal Microscopy

SCC-15 and SCC-25 cells (5.0 × 10^3^ cells) were seeded on a glass-bottom dish (Corning, USA) and cultured with cisplatin (1 *μ*g/mL) for 24, 48, and 72 h and with different concentrations of cisplatin (0, 1, 2, and 4 *μ*g/mL) for 24 h. Regular media was used as the control. Primary antibodies to *β*-catenin (8480S, 1 : 100, Cell Signaling Technology) were added to the cells and incubated at 4°C overnight. After washing, FITC-conjugated goat anti-rabbit IgG (1 : 1000, Cell Signaling Technology) was added and incubated at room temperature in the dark for 2 h and then covered with Prolong® Gold Antifade Reagent and DAPI for 10 minutes at room temperature (#8961, 4,6-diamidino-2-phenylindole; Sigma). Confocal images were taken with a Zeiss LSM 510 laser scanning confocal microscope (Carl Zeiss, Jena, Germany).

### 2.6. Reverse Transcription-Quantitative PCR (RT-qPCR)

Total RNA was isolated from the OSCC cell lines using TriPure (Roche Molecular Biochemicals, Mannheim, Germany) according to the manufacturer's instructions. 1 *μ*g of total RNA for one 20 *μ*L reaction volume was reverse transcribed to cDNA using the transcriptor first-strand cDNA synthesis kit (Roche Molecular Biochemicals, Mannheim, Germany) according to the manufacturer's instructions. The first-strand cDNA synthesis was performed by the use of Applied Biosystems® Veriti PCR (Veriti, Thermo Fisher, USA). qPCR was performed using a LightCycler® 480 SYBR Green I Master (Roche Diagnostics, Germany), following thermal cycling parameters: preincubation at 95°C for 5 min, followed by 40 cycles of amplification (95°C for 10 s, 60°C for 20 s, and 72°C for 30 s), melting curve (95°C for 5 s, 65°C for 60 s, and 97°C-), and cooling (40°C for 10 s). Expression levels were analyzed using the ΔΔCq method [25], and gene expression was normalized to the internal control, glyceraldehyde 3-phosphate dehydrogenase. The primers are shown in Table 1. All samples were analyzed in triplicate.

### 2.7. Western Blotting

SCC-25, SCC-15, and the cells with *β*-catenin knockin and knockdown were lysed with RIPA buffer (Beyotime Biotechnology, China) and nuclear fractions of SCC-25 and SCC-15 cells were isolated by the use of Cellytic*™* Nuclear*™* Extraction Kit (NXTRACT, Sigma, USA) to detect the cellular localization of *β*-catenin. Protein concentrations were quantified by the use of BCA Protein Assay Kit (Beyotime Biotechnology, China). Western blotting was performed according to standard protocols. 40~60 *μ*g total protein extract was separated by SDS-PAGE on a 8%–10% gradient gel and then transferred onto a PVDF membrane (Millipore, Billerica, MA, USA) for 1 to 2 h. The membrane was blocked in 5% nonfat dry milk at room temperature for 1 hour and incubated overnight at 4°C with the following antibodies: anti-MRP-1 (ab32574, 1 : 1000, Abcam), anti-P-gp (ab3366, 1 : 1000, Abcam), anti-C-myc (5605S, 1 : 1000, Cell Signaling Technology), anti-GSK-3*β* (9315S, 1 : 1000, Cell Signaling Technology), anti-Bcl-2 (2870S, 1 : 1000, Cell Signaling Technology), anti-*β*-catenin (9582S, 1 : 1000, Cell Signaling Technology), anti-*β*-actin (4970S, 1 : 1000, Cell Signaling Technology), and anti-histone (9715, 1 : 1000, Cell Signaling Technology). The secondary antibody was horseradish peroxidase-conjugated anti-mouse (7076P2, 1 : 1000, Cell Signaling Technology) and anti-rabbit (7074S, 1 : 1000, Cell Signaling Technology). The membrane was then incubated with HRP-linked secondary antibodies (1 : 1000) at room temperature for 2 h. Membranes were analyzed by chemiluminescence using an ECL system (Invitrogen, China) and bands were visualized using FluorChem Q (Alpha Innotech, Sunnyvale, CA, USA). All experiments were repeated at least three times.

### 2.8. Animal Study

Male BALB/c-nu nude mice (*n* = 24; 3-4 weeks old) from the Laboratory Animal Center of Sun Yat-sen University were used in the study. Mice were maintained in a temperature controlled on a 12-h light-dark cycle, with free access to food and water at 22°C. For the overexpression of *β*-catenin, a total of 1 × 10^7^ SCC-25 cells with CTNNB1 knockin were injected in the left posterior flanks, and the right anterior flanks were used as the negative control. For the decreased expression of *β*-catenin, a total of 1 × 10^7^ SCC-25 cells with CTNNB1 knockdown were injected in the left posterior flanks, and the right anterior flanks were used as the negative control. The tumor size was measured using Vernier calipers. Tumor volume was calculated by the following formula: volume = 0.5 × length × width^2^ (length: longest diameter in mm, width: the shortest diameter in mm). When the tumor had grown to an average volume of 100 mm^3^ (after about 14 days), the mice were given an injection of cisplatin (4 mg/kg) through the tail vein at 3-day intervals for five times. The tumor size was monitored every 3 days after inoculation. The animals were then sacrificed under anesthesia with an overdose of pentobarbital sodium (50 mg/kg, 1063180500, Merck, Germany) and individual tumor volumes were calculated. All animal procedures were approved by the Sun Yat-sen University Medical Experimental Animal Care Commission.

### 2.9. Statistical Analysis

All data were expressed as the means ± SD and were collected from at least three replicates per experiment. Data were analyzed using Student's *t*-test using SPSS 19.0 statistical software. A value of *P* < 0.05 was considered to be statistically significant.

## 3. Results

### 3.1. Cisplatin Increases the Expression Level of *β*-Catenin and Stimulates Nuclear Translocation in OSCC Cell Lines

**Figure 1:**
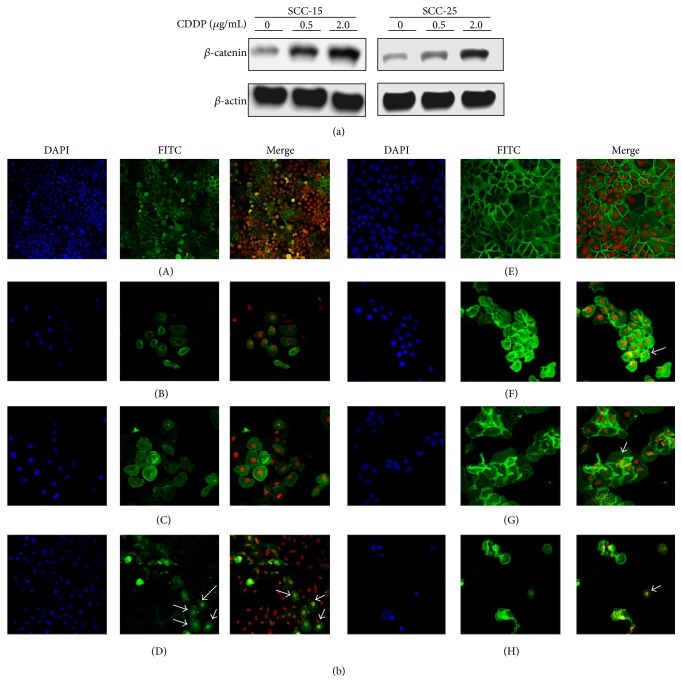
Cisplatin increased *β*-catenin expression levels and mediated nuclear translocation in OSCC cell lines. (a) Western blot analysis showed protein expression levels of *β*-catenin were correlated with the cisplatin treatment. (b) Confocal microscopy showed cisplatin stimulated nuclear translocation of *β*-catenin (indicated by the arrow). (A–D) SCC-25 cells treated with cisplatin (0, 1, 2, and 4 *μ*g/mL) for 24 h; (E–H) SCC-15 cells treated with cisplatin (1 *μ*g/mL) for 0, 24, 48, and 72 h (magnification: 200х).

We found the expression levels of *β*-catenin were increased in cisplatin-treated OSCC cell lines, and this increase was significantly correlated with the concentration of cisplatin (Figure 1(a)). To investigate whether cisplatin caused *β*-catenin to translocate to the nucleus, we examined the OSCC cells by confocal microscopy and by Western blotting. The cisplatin-treated cells showed higher expression levels of *β*-catenin in the nucleus (Figure 1(b)), whereas expression of *β*-catenin in the control groups was found mostly on the cell membrane. In the cisplatin treatment groups, more *β*-catenin translocated from the membrane to nucleus as the concentration of cisplatin increased (Figure 1(c)).

### 3.2. Knockdown of *β*-Catenin Suppresses Cell Proliferation and Enhances Sensitivity to Cisplatin in OSCC

**Figure 2:**
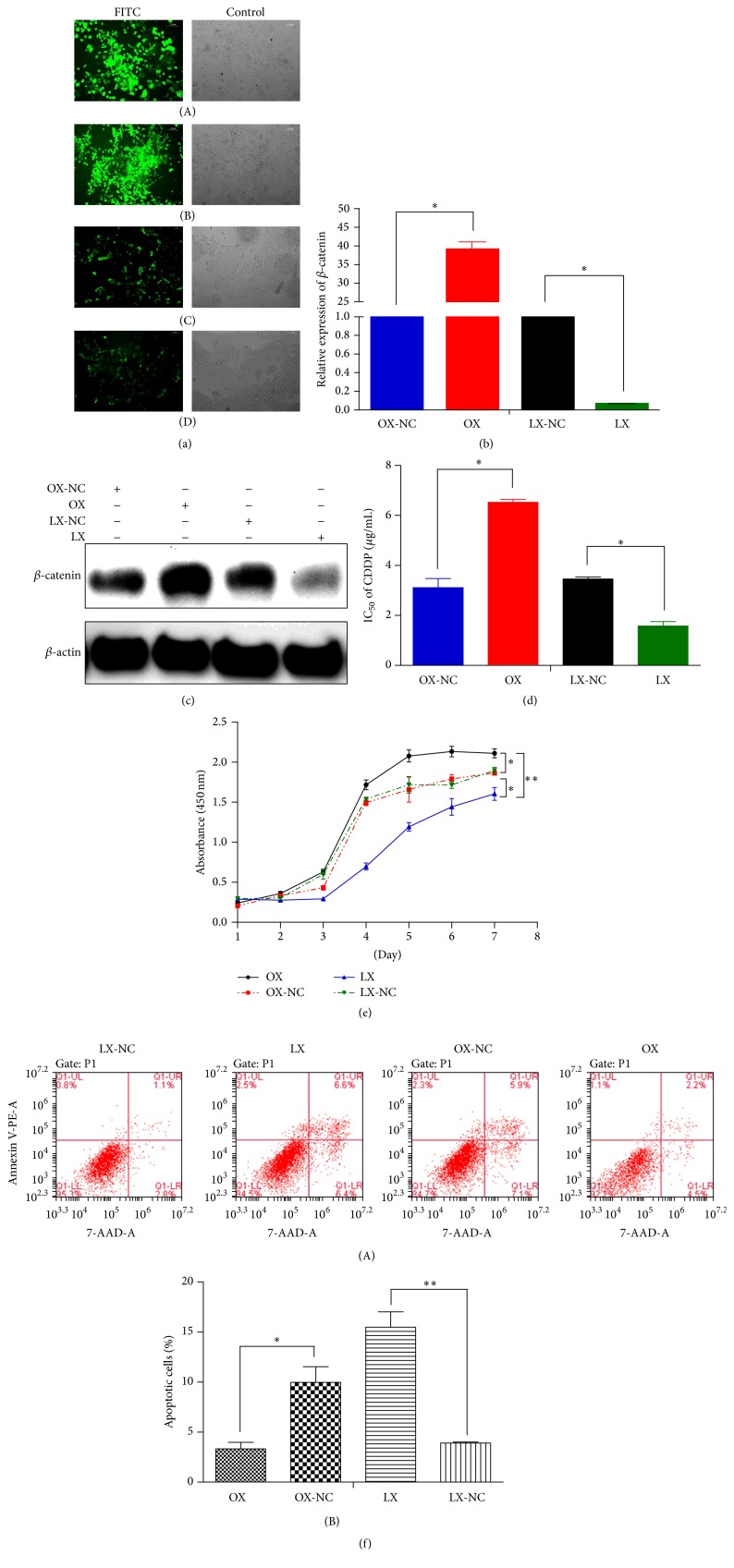
*β*-catenin knockdown promoted apoptosis and sensitivity to cisplatin but suppressed proliferation in SCC-25 cells. (a) Transfection efficiency of the cells was evaluated by the fluorescence microscopy (A: OX cells, B: OX-NC cells, C: LX cells, and D: LX-NC cells). (b) Gene expression of *β*-catenin was confirmed by PCR. (c) Protein expression of *β*-catenin was confirmed by Western blot analysis. (d) *β*-catenin knockdown caused a significant decrease in cisplatin IC_50_ of SCC-25 cells. (e) CCK-8 assay showed *β*-catenin knockdown suppressed proliferation in SCC-25 cells. (f) Flow cytometry analysis showed *β*-catenin knockdown promoted apoptosis (OX: overexpression, OX-NC: overexpression-negative control, LX: low expression, and LX-NC: low expression-negative control). ^*∗*^ *P* < 0.05; ^*∗∗*^ *P* < 0.01 (magnification: 100х).

We established stable cells lines with CTNNB1 knockin and knockdown by lentiviral transfection. Transfection efficiency was evaluated by fluorescence microscopy (Figure 2(a)). The efficiency of CTNNB1 knockin and knockdown was confirmed by PCR (Figure 2(b)) and Western blot analysis (Figure 2(c)). The IC_50_ values of cisplatin in the SCC-25 cells with CTNNB1 knockin and knockdown were evaluated by CCK-8 assays. Our data showed that overexpression of *β*-catenin increased IC_50_ of cisplatin, whereas decreased expression of *β*-catenin led to decreased IC_50_ (Figure 2(d)). The proliferation and apoptosis assays showed cell proliferation and survival were significantly decreased (*P* < 0.05) in SCC-25 cells with CTNNB1 knockdown compared to the controls. Correspondingly, cell proliferation and survival were significantly increased in SCC-25 cells with CTNNB1 knockin after treatment with cisplatin (Figures 2(e) and 2(f)).

### 3.3. MRP-1, P-gp, Bcl-2, and Wnt Signaling Pathway Are Involved in *β*-Catenin-Mediated Cisplatin Chemoresistance

**Figure 3:**
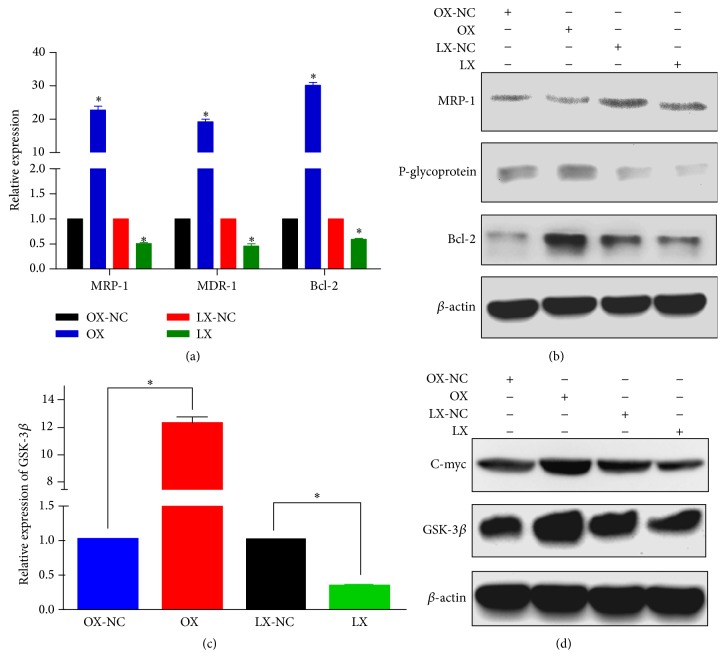
Overexpression of *β*-catenin increased the chemoresistance of SCC-25 cells to cisplatin and promoted cell survival. (a) Overexpression of *β*-catenin increased the expression levels of Bcl-2, MRP-1, and MDR-1 compared to the controls. (b) Western blot analysis of the protein expression levels of Bcl-2, MRP-1, and MDR-1. (c) Expression of *β*-catenin was involved in the Wnt signaling pathway by regulation of GSK-3*β* and C-myc. (d) Western blot analysis of the protein expression levels of GSK-3*β* and C-myc. ^*∗*^ *P* < 0.05.

To determine the effects of *β*-catenin on chemotherapy, we examined the associated resistance proteins and resistance genes by Western blotting and RT-PCR. We found increased expression levels of Bcl-2, MRP-1, and MDR-1 in SCC-25 cells with CTNNB1 knockin compared with the controls (Figure 3(a)). Conversely, we found decreased levels of Bcl-2, MRP-1, and MDR-1 in the SCC-25 cells with CTNNB1 knockdown. Moreover, Western blot analysis showed increased protein expression levels of Bcl-2, MRP-1, and P-gp in SCC-25 cells with CTNNB1 knockin compared with the controls. Expression levels of these proteins were decreased in the SCC-25 cells with CTNNB1 knockdown (Figure 3(b)). Therefore, overexpression of *β*-catenin in SCC-25 cells may increase their resistance to cisplatin therapy, whereas decreased expression of *β*-catenin in SCC-25 cells may enhance their sensitivity to cisplatin. We further investigated the mechanisms involved in the chemoresistance of OSCC cells to cisplatin. We analyzed the expression levels of GSK-3*β* and C-myc in the two stable expression cell lines by RT-PCR (Figure 3(c)) and by Western blot analysis (Figure 3(d)) of the whole cell lysates. We found an increase in the expression levels of GSK-3*β* and C-myc in the SCC-25 cells with CTNNB1 knockin compared with the controls. Conversely, we found a significant reduction in the expression levels of GSK-3*β* and C-myc in SCC-25 cells with CTNNB1 knockdown compared with the controls. Thus, *β*-catenin could be involved in the activation of Wnt/*β*-catenin signaling pathway through the regulation of GSK-3*β* and C-myc.

### 3.4. Decreased Expression of *β*-Catenin in SCC-25 Cells Increases Cisplatin Sensitivity and Suppresses Tumor Formation in BALB/c Nude Mice

**Figure 4:**
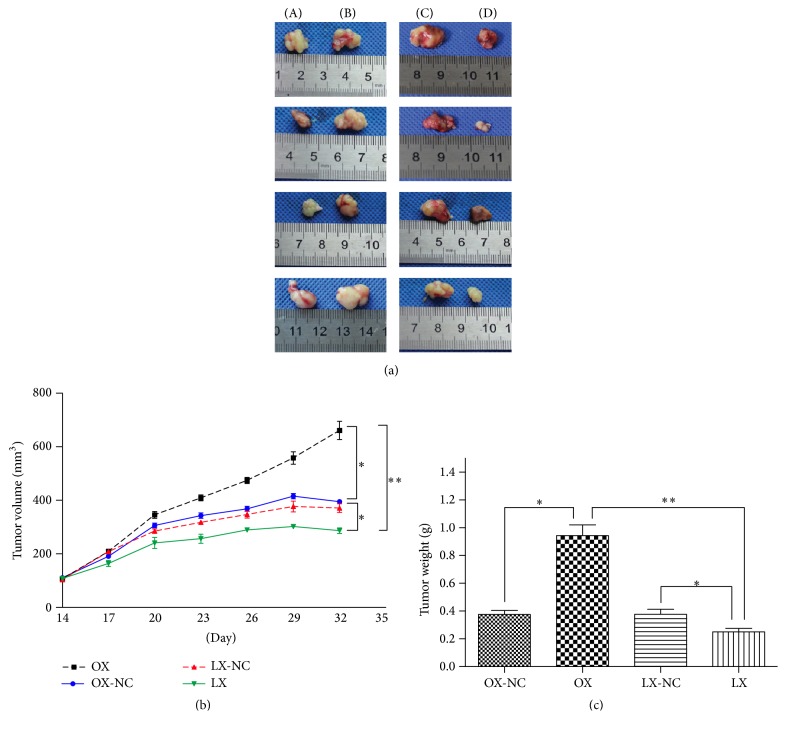
*β*-catenin knockdown increased sensitivity to cisplatin in vivo. (a) Photographs of tumors (A: OX-NC, B: OX, C: LX-NC, and D: LX). (b) Tumor sizes were measured from day 14 until mice were killed. When tumor volumes reached 100 to 150 mm^3^, mice were injected with 4 mg/kg cisplatin through the tail vein. (c) Tumor weight was measured at the end of the experiment. The tumor weight was significantly reduced in mice treated with OSCC cells with *β*-catenin knockdown after cisplatin treatment, whereas the tumor size continuously increased with the overexpression of *β*-catenin indicating promoted tumor growth. ^*∗*^ *P* < 0.05; ^*∗∗*^ *P* < 0.01.

To evaluate the effect of *β*-catenin on cisplatin chemotherapy, SCC-25 cells with CTNNB1 knockin or knockdown were implanted subcutaneously into left posterior flanks of BALB/c nude mice. Individual tumors from mice treated with cisplatin were collected and weighed. The average tumor volume of the CTNNB1 knockdown group was significantly smaller than those from the control group (Figure 4(a)), which showed that the tumor formation was suppressed in the cisplatin-treated group with CTNNB1 knockdown (Figure 4(b)). In addition, the average tumor weight was markedly reduced compared with the *β*-catenin overexpression or control groups (Figure 4(c)). Conversely, the overexpression of *β*-catenin in the cisplatin-treated mice promoted tumor formation with continuous increase of the tumor size compared to the other groups, which indicated overexpression of *β*-catenin promoted chemoresistance. These results provide evidence that knockdown of *β*-catenin in OSCC cells may enhance their sensitivity to cisplatin and reduce the tumor growth in vivo.

## 4. Discussion

Recent accumulating evidence has supported the notion that Wnt/*β*-catenin signaling is associated with resistance to chemotherapy in several cancers [25, 26]. Our findings showed higher expression levels of *β*-catenin were found in specimens from patients who had received cisplatin chemotherapy compared to those who had not received chemotherapy [23], which was confirmed in human OSCC cell lines treated with cisplatin. In these treatment groups, the expression levels of *β*-catenin were found to be increased in a concentration- and time-dependent manner with the cisplatin treatment. We also observed nuclear translocation of *β*-catenin in cisplatin-treated groups by confocal microscopy and Western blot analysis. These results provide strong evidence that *β*-catenin plays an essential role in cisplatin-mediated resistance in OSCC. However, the molecular mechanisms of drug resistance and the relationship with abnormal expression of *β*-catenin in OSCC have not been clearly defined.

Several mechanisms of cisplatin resistance have been extensively reported [27], involving reduced cisplatin uptake and accumulation, activation of DNA repair mechanisms and decreased DNA mismatch repair [28], apoptosis inhibition and reduced apoptotic response [29], and cell signaling molecules and pathways [30]. Our results showed overexpression of *β*-catenin in OSCC cells led to a higher cisplatin IC_50_ after 24 h of treatment (*P* < 0.05) compared to reduced expression of *β*-catenin in OSCC cells. *β*-catenin was reported to be involved in chemoresistance/radioresistance in colon cancer cells [31]. Conversely, we found decreased expression of *β*-catenin in OSCC cells suppressed cell proliferation and promoted apoptosis, making the cells more sensitive to cisplatin, which was also confirmed in our animal study results. *β*-catenin is an important adhesion molecule and a key regulator in Wnt signaling pathway. Wnt/*β*-catenin signaling pathway has been shown to be involved in cisplatin resistance through the regulation of *β*-catenin. Upregulation of *β*-catenin expression in SCC-25 cells led to an overexpression of GSK-3*β* and C-myc [32], whereas downregulation of *β*-catenin expression led to lower expression levels of GSK-3*β* and C-myc. Both GSK-3*β* and *β*-catenin are two key regulators in Wnt/*β*-catenin signaling pathway involved in the regulation of cell growth and adhesion. In the absence of Wnt signaling, *β*-catenin is phosphorylated by GSK-3*β* and targeted for degradation [33]. Activation of Wnt signaling prevents *β*-catenin from being phosphorylated by GSK-3*β*, resulting in its nuclear translocation [34, 35]. In our present study, *β*-catenin translocated and accumulated in the nucleus in OSCC cells treated with cisplatin. Accumulation of nuclear *β*-catenin and the subsequent binding to Tcf transcription factors [36] promoted the overexpression of downstream target genes, such as C-myc, which we also observed in our results. Moreover, C-myc has been reported to be overexpressed in cisplatin resistance [32]. The abnormal expression of downstream target genes can lead to abnormal proliferation of tumor cells and the increased ability of cancer cells to evade apoptosis [37] allowing them to develop cisplatin resistance [38]. Bcl-2 is an antiapoptotic protein of the Bcl-2 family that is involved in chemotherapy [39, 40]. We found increased expression levels of Bcl-2 in OSCC cells overexpressing *β*-catenin. Moreover, evidence suggests that overexpression of Bcl-2 in tumor cells can result in their escape from cell apoptosis and resistance to anticancer drugs [41]. Conversely, it was reported that low expression levels of Bcl-2 could promote apoptosis of OSCC cells [42], which was consistent with our results. In addition, we found overexpression of *β*-catenin in SCC-25 cells led to higher expression levels of P-gp and MRP-1, whereas the reverse was true with reduced expression of *β*-catenin compared with the control cells. Substantial evidence suggests that P-gp [43] and MRP-1 [44] are associated with multidrug resistance in several types of advanced cancer. Elevated levels of P-gp and MRP-1 have been reported in cancer cells showing an acquired multidrug-resistant phenotype following chemotherapy, whereas low levels of P-gp and MRP-1 were observed in cancer cells before chemotherapy [45, 46].

In conclusion, our findings revealed that overexpression of *β*-catenin was associated with cisplatin resistance in OSCC cells and that reduced expression of *β*-catenin could confer sensitivity to cisplatin resulting in better treatment efficacy. However, the precise molecular mechanisms and clinical significance of our findings need to be further investigated. Our results demonstrated that *β*-catenin might play important roles in cisplatin resistance in OSCC through the regulatory mechanisms of Wnt/*β*-catenin signaling pathway. Therefore, a thorough understanding of molecular mechanism involving *β*-catenin would enable the development of novel strategies to overcome possible drug resistance.

## Figures and Tables

**Table 1:** Sequences of oligonucleotides used in RT-PCR analysis.

Gene	Accession number	Forward primer	Reverse primer
*β*-catenin	NM_001098209	CATCTACACAGTTTGATGCTGCT	GCAGTTTTGTCAGTTCAGGGA
GSK-3*β*	NM_001146156	GGCAGCATGAAAGTTAGCAGA	GGCGACCAGTTCTCCTGAATC
Bcl-2	NM_000633	GGTGGGGTCATGTGTGTGG	CGGTTCAGGTACTCAGTCATCC
MRP-1	NM_004996	GTCGGGGCATATTCCTGGC	CTGAAGACTGAACTCCCTTCCT
MDR-1	NM_000927	GGGAGCTTAACACCCGACTTA	GCCAAAATCACAAGGGTTAGCTT
GAPDH	NM_001256799	TCCAAAATCAAGTGGGGCGA	AAATGAGCCCCAGCCTTCTC
siRNA3-homo-1302	AGGTGCTATCTGTCTGCTCTA		
Negative	TTCTCCGAACGTGTCACGTTTC		
